# The profile of selected single nucleotide polymorphisms in patients with hypertension and heart failure with preserved and mid-range ejection fraction

**DOI:** 10.1038/s41598-017-09564-9

**Published:** 2017-08-21

**Authors:** Agata Bielecka-Dabrowa, Agata Sakowicz, Tadeusz Pietrucha, Małgorzata Misztal, Piotr Chruściel, Jacek Rysz, Maciej Banach

**Affiliations:** 1 Department of Hypertension, Chair of Nephrology and Hypertension, Medical University of Lodz, Zeromskiego 113, 90-549 Lodz, Poland; 2 Department of Medical Biotechnology, Medical University of Lodz, Zeligowskiego 7/9, Lodz, Poland; 3 Chair of Statistical Methods, Faculty of Economics and Sociology, University of Lodz; Rewolucji 1905/41, 90-214 Lodz, Poland; 4 Department of Nephrology, Hypertension and Family Medicine, Chair of Nephrology and Hypertension, Medical University of Lodz, Zeromskiego 113, 90-549 Lodz, Poland

## Abstract

The study aimed to assess the clinical significance of selected single nucleotide polymorphisms (SNPs) in patients with diastolic heart failure (HF): inflammation [-174 G/C Interleukin -6 (IL-6) rs1800795, tumor necrosis factor (TNF)-608 G/A rs1800629], fibrosis [Arg25Pro transforming growth factor β (TGF β) rs1800471], endothelial function [-786 T/C nitric oxide synthase (NOS) rs2070744], glucose and lipid metabolism [Pro12Ala peroxisome proliferator activated receptor (PPAR)γ rs1801282], and vitamin D metabolism [cytochrome P450 27B1 (CYP27B1) C-1260A].110 patients with HF with preserved and mid-range ejection fraction (HFpEF and HFmrEF) were recruited. GG homozygotes in 174 G/C of IL6 polymorphism are characterized by higher values of estimated glomerular filtration rate based on the study Modification of Diet in Renal Disease (eGFR MDRD) and C allele in the NOS polymorphism and AA profile in C-1260A of CYP27B1 polymorphism correlated with a lower eGFR (MDRD). In multivariate analysis the CG genotype for 174 G/C of IL-6 and allele A in C-1260A of CYP27B1 are the only SNPs independently associated with worse course of HFpEF and HFmrEF. These data confirm the importance of the selected SNPs in aggravation and complications of hypertension.

## Introduction

Left ventricular (LV) diastolic dysfunction is an increasingly prevalent disease process in hypertensive patients^1^. Hypertension is the most prevalent comorbidity in heart failure (HF) and can lead to different activated pathophysiological mechanisms in heart failure with preserved and mid-range reduced ejection fraction (HFpEF and HFmrEF).

Current studies suggest that natriuretic peptides have the potential to improve the establishment of diagnosis of early HFpEF but they still have significant limitations. There are still doubts whether the cut-off points of N-terminal pro brain natriuretic peptide (NT-proBNP) and brain natriuretic peptide (BNP) in diagnosis and monitoring of diastolic HF should be the same for the whole HF spectrum^2^.

In HFpEF, extracardiac comorbidities such as metabolic risk, arterial hypertension, and renal insufficiency drive left ventricular remodeling and dysfunction through systemic inflammation and coronary microvascular endothelial dysfunction. The latter affects left ventricular diastolic dysfunction through macrophage infiltration, resulting in interstitial fibrosis, and through altered paracrine signaling to cardiomyocytes, which become hypertrophied and stiff because of low nitric oxide and cyclic guanosine monophosphate. Systemic inflammation also affects other organs such as lungs, skeletal muscle, and kidneys, leading, respectively, to pulmonary hypertension, muscle weakness, and sodium retention^3^.

The new therapies should be targeted towards specific HF phenotypes, instead of the ‘one-size-fits-all’ approach, which has not been successful in clinical HFpEF. Unless the structural and biological determinants of the failing heart are deeply understood, it will be impossible to differentiate HFpEF patients appropriately, identify subtle myocardial abnormalities, and finally reverse abnormal cardiac function. The identification of specific structural cardiac HFpEF phenotypes may represent one way to differentiate subgroups of HFpEF patients with specific therapeutic targets. The use of selective biomarkers in diastolic HF may help to distinguish pathophysiological mechanisms of HFpEF and HFmrEF. Because of phenotypic diversity in HFpEF and HFmrEF, personalized therapeutic strategies are proposed^1–4^. In this study we assessed the clinical significance of the single nucleotide polymorphisms reflecting different pathophysiological pathways in diastolic HF as complications of hypertension: inflammation [-174 G/C interleukin -6 (IL-6) rs1800795, tumor necrosis factor (TNF)-608 G/A rs1800629], fibrosis [Arg25Pro transforming growth factor β (TGF β) TGFβ rs1800471], endothelial function [-786 T/C nitric oxide synthase (NOS) rs2070744), glucose and lipid metabolism [Pro12Ala peroxisome proliferator-activated receptor gamma (PPARγ) rs1801282), and vitamin D metabolism [cytochrome P450 27B1 (CYP27B1) C-1260A]. Our polymorphisms selection we was based on careful analysis of literature in the context of diastolic HF potential patomechanisms and pathogenetic mechanisms^2–4^.

## Results

### General Characteristics of patients

The patients’ characteristics are presented in Tables 1 and 2. Patients in the assessed group were on average aged 63 ± 11 years (min-max: 30–85 years) with well-controlled blood pressure, and 69% were male. In our study in a Caucasian population of hypertensive patients the frequency of the GG genotype was the highest in 174 G/C of IL-6 and TNF-308 G/A rs1800629. We did not observe the GG genotype in TGF beta 915 G > C (Arg25Pro) rs1800471; patients presented mainly CC genotype in this polymorphism. Only 5.5% in C-1260A of CYP27B1 were AA homozygotes and 1% in NOS3–786 C/T rs2070744 were CC homozygotes; in these SNPs there was the highest number of heterozygotes (Table 3). In PPAR gamma Pro12Ala (34 C/G) individuals carried mainly the CC genotype and the least frequently the homozygous genotype GG (Table 2).

**Table 1:** Characteristics of patients, standard echocardiographic parameters and biochemical biomarkers in HF patients – quantitative and categorical variables.

Parameter	Median (Q25-Q75)	Parameter	Number of patients (%)
Age (years)	65 (58–71)	Gender (male)	76 (69)
BMI kg/m^2^	28 (25–30)	Smoking	5 (5)
eGFR (MDRD) (ml/min/1.73 m²)	88 (64–92)	Metabolic syndrome	43 (39)
		Heart failure acc. to NYHA	
Systolic BP mmHg	130 (120–140)	I	39 (36)
		II	43 (39)
		III	28 (25)
		Mitral incompetence degree	
Diastolic BP mmHg	80 (70–80)	0	16 (14)
		I	64 (59)
		II	22 (20)
		III	8 (7)
		Tricuspid incompetence degree	
HR	70 (65–78)	0	27 (25.2)
		I	61 (57.5)
		II	16 (15)
		III	2 (2)
Hemoglobin g/dl	14 (13–15.1)	Lung congestion	9 (8)
LVEDD (mm)	55 (50.5–63)	Leg edema	10 (9)
LVESD (mm)	38 (32–50)	Diabetes mellitus or abnormal glucose level	18 (16)
LVEF (%)	48 (40–60)	Statins	61 (55)
LA (mm)	41 (36–45)	Insulin	8 (7)
E/E’ ratio	15 (14–16)	Diuretics	72 (66)
RVDD (mm)	28 (25–31)	Beta-blockers	96 (88)
TAPSE (mm)	24 (21–27)	Spironolactone/eplerenone	58 (53)
LV mass (g)	195.5 (169.5–234.5)	Acetylsalicylic acid	53 (48)
LVMI (g/m2)	104 (87–115)	ACE inhibitors	79 (72)
CRP mg/l	1.7 (1.1–3.89)	ARBs	26 (23)
TNF alpha (pg/ml)	19.9 (6.5–31)	CCB	20 (18)
IL 6 (pg/ml)	18 (14–21)	Digoxin	8 (7)
IL1R1(ng/ml)	0.28 (0.13–0.54)	Parox AF	15 (13)
TGF beta 1 (ng/ml)	8.3 (5.05–10.8)		
Syndecan 4 (ng/ml)	1.5 (0.69–3.1)		
Cardiotrophin (pg/ml)	116.2 (43–222.2)		
NT-proBNP (pg/ml)	195 (130–275)		
Cystatin C (mg/l)	0.96 (0.8–1.28)		
Lipocalin-2 NGAL (ng/ml)	47.6 (31.6–65.8)		
Uric acid (mg/dl)	6 (5.1–7)		

ABBREVIATIONS: BMI, body mass index; eGFR (MDRD), glomerular filtration rate on the basis of the study Modification of Diet in Renal Disease; BP, blood pressure; HR, heart rate; LVEDD, left ventricular end-diastolic diameter; LVESD, left ventricular end-systolic diameter; LVEF, left ventricular ejection fraction; LA, left atrial diameter; E/E’, ratio of early mitral diastolic inflow velocity to early diastolic mitral annular velocity; RVDD, right ventricular diastolic diameter; TAPSE, tricuspid annular plane systolic excursion; LVMI, left ventricular mass index; NYHA, New York Heart Association classification of heart failure; ACE inhibitors, angiotensin-converting enzyme inhibitors; ARBs, angiotensin receptor blockers; CCB, calcium channel blocker; TGF beta 1, ***transforming growth factor beta 1;*** NT-proBNP, N-terminal pro-brain natriuretic peptide; NGAL, neutrophil gelatinase-associated lipocalin; TNF alpha, tumor necrosis factor alpha; IL1R1, interleukin 1 receptor, type I; Parox AF, paroxysmal atrial fibrillation.

**Table 2:** Profiles of genetic biomarkers in HF patients.

SNP	Number of patients (%)
174 G/C of IL-6	
GG	78(71)
CC	11(10)
GC	21(19)
TNF-308 G/A rs1800629	
GA	24(22)
GG	85(77)
AA	1(1)
PPAR gamma Pro12Ala (34 C/G)	
CC	75(68)
CG	31(28)
GG	4(4)
TGF beta 915 G > C (Arg25Pro) rs1800471	
CC	88(80)
CG	22(20)
C-1260A of CYP27B1	
CA	60(54,5)
CC	44(40)
AA	6(5,5)
NOS3 -786 C/T rs2070744	
CT	62(56)
TT	47(43)
CC	1(1)

ABBREVIATIONS: SNP, single nucleotide polymorphism; IL-6, interleukin 6; TNF alpha, tumor necrosis factor alpha; PPAR gamma, peroxisome proliferator-activated receptor gamma; TGF beta, t***ransforming growth factor beta;*** CYP27B1, cytochrome P450, family 27, subfamily B, polypeptide 1; NOS3, nitric oxide synthase 3.

**Table 3:** The significant differences in selected polymorphisms.

174 G/C of IL-6 polymorphism			
Parameters with significant differences	Non-GG profile	GG profile	p
	N = 32 (29%)	N = 78 (71%)	
eGFR MDRD (ml/min/1.73 m²)	66.4 (53–88)	89 (75–93)	0.014
S	7 (6.1–8.4)	8.8 (6.9–10.5)	0.033
**TNF-308 G/A rs1800629 polymorphism**			
**Parameters with significant differences**	**Non-A allele**	**A allele**	**p**
	**N = 85 (77%)**	**N = 25 (23%)**	
Systolic BP mmHg	130 (120–140)	120 (110–130)	0.007
Paroxysmal AF	7 (8.4%)	8 (32%)	0.006
**TGF beta 915 G > C** (**Arg25Pro**) **rs1800471 polymorphism**			
**Parameters with significant differences**	**CC profile**	**CG profile**	**p**
	**N = 88 (80%)**	**N = 22 (20%)**	
LV mass	201 (173–260)	170 (149–196)	0.045
TNF alpha (pg/ml)	21 (10–31)	14 (2.9–21)	0.042
**NOS3–786 C/T rs2070744 polymorphism**			
**Parameters with significant differences**	**Non-C allelle**	**C allelle**	**p**
	**N = 47 (43%)**	**N = 63 (57%)**	
Class acc. NYHA	2 (1–2)	2 (1–3)	0.039
Systolic BP mmHg	130 (125–140)	130 (120–135)	0.034
Diastolic BP mmHg	80 (80–90)	80 (70–80)	0.002
Cystatin C (mg/l)	0.90 (0.62–1.17)	1.01 (0.88–1.3)	0.022
eGFR (MDRD) (ml/min/1.73 m²)	89 (79–93)	77 (59–91)	0.020
LVEF (%)	56 (40–70)	45 (40–67)	0.007

Presented values: median (Q25-Q75). ABBREVIATIONS: eGFR MDRD, estimated glomerular filtration rate on the basis of the study Modification of Diet in Renal Disease; S’, systolic mitral annular velocity; BP, blood pressure; AF, atrial fibrillation; LV, left ventricular; TNF, tumor necrosis factor; NYHA, New York Heart Association; LVEF, left ventricular ejection fraction.

### Significant correlations of selected polymorphisms

There were no significant changes between HF patients with and without the G allele. GG homozygotes in 174 G/C of IL6 polymorphism are characterized by higher values of estimated glomerular filtration rate based on the study Modification of Diet in Renal Disease (eGFR MDRD) (ml/min/1.73 m²)] and systolic mitral annular velocity (S). There were higher eGFR (p = 0.01) and higher S (p = 0.03) for GG genotype in hypertensive patients with HF –Table 3.

HF patients with the A allele in TNF-308 G/A rs1800629 polymorphism had lower systolic blood pressure and higher incidence of paroxysmal atrial fibrillation –Table 3.

There were no differences between patients with HF with and without the G allele in PPAR gamma polymorphism.

Patients with HF and CC profile of TGF beta polymorphism had higher LV mass and a higher level of TNF alpha in plasma –Table 3.

Patients with the C allele in NOS polymorphism had a higher class according to the New York Heart Association (NYHA) classification (p = 0.04), lower blood pressure (p = 0.03), a higher level of cystatin C (p = 0.02), lower eGFR MDRD (p = 0.02), and lower left ventricular ejection fraction (LVEF) (p = 0.007) compared to patients without the C allele -Table 3.

Patients with AA profile in C-1260A of CYP27B1 polymorphism had lower values of eGFR (MDRD) (p = 0.02) and higher levels of uric acid (p = 0.03) compared to the CA and CC profile. There were no significant differences according to CC vs non-CC profile in CYP 27 polymorphism –Table 4.

**Table 4:** The significant differences in CYP27B1 -1260C/A rs10877012 polymorphism.

CYP27B1 -1260C/A rs10877012 polymorphism					
Parameters with significant differences	CA profile	CC profile	AA profile	p-ANOVA	Comparison between groups–significant differences
	N = 60 (55%)	N = 44 (40%)	N = 6 (5%)		
Uric acid (mg/dl)	5.9 (4.8–6.7)	6.5 (5.6–7.5)	9 (5.1–10.3)	0.032	AA vs CA – p = 0.01
					AA vs CC – p = 0.08
eGFR (MDRD) (ml/min/1.73 m²)	88 (75–93)	88 (64–92)	53.5 (43.5–60)	0.022	AA vs CA – p = 0.02
					AA vs CC – p = 0.06

Presented values: median (Q25-Q75). ABBREVIATIONS: eGFR MDRD – estimated glomerular filtration rate on the basis of the study Modification of Diet in Renal Disease.

### Multivariate logistic regression analysis

Logistic regression analysis was used to identify the risk factors for a worse HF course based on NYHA classification scale, which is useful in assessing the stage of HF based on how much the patient is limited during physical activity.

Variables significant in univariate comparisons (p < 0.05) [age, systolic blood pressure (BP), transforming growth factor beta 1 (TGF beta 1), syndecan 4, NT-proBNP, cystatin C, interleukin 1 receptor, type I (IL1R1), eGFR MDRD, interleukin 6 (IL6) level, presence of C allele in NOS3 -786 C/T rs2070744 polymorphism, CG genotype for 174 G/C of IL-6, presence of A allele in C-1260A of CYP27B1 polymorphism) were included in the multivariate logistic regression model – the stepwise logistic regression to identify the set of statistically significant risk factors of symptomatic HF (classes II and III according to the NYHA classification).

In multivariate analysis CG genotype for 174 G/C of IL-6 (odds ratio OR = 7.5; 95% CI: 1.1–50; *p* = 0.03), allele A in C-1260A of CYP27B1 (OR = 4.3; 95%CI: 1.3–13.8; *p* = 0.01), lower GFR (MDRD) (OR = 0.96; 95%CI: 0.92–1.0; *p* = 0.05), higher level of cystatin C (OR = 6.6; 95%CI: 1.2–35.2; *p* = 0.02) and lower level of TGF beta 1 (OR = 0.96; 95%CI: 0.57–0.84; *p* = 0.001) were independent risk factors of worse course of HF (Table 5).

**Table 5:** Stepwise logistic regression assessing factors influencing limitations during activity in NYHA classification.

Variable	OR	95%CI for OR		*p-value*
		Lower limit	Upper limit	
Constant	x	x	x	0.091
TGF beta 1	0.69	0.57	0.84	< 0.001
Cystatin C	6.60	1.23	35.2	0.027
eGFR (MDRD)	0.96	0.92	1.0	0.050
CG genotype for 174 G/C of IL-6	7.53	1.13	50.0	0.037
A allele in C-1260A of CYP27B1	4.30	1.33	13.89	0.014

Statistical analysis with stepwise logistic regression. ABBREVIATIONS: OR – odds ratio; CI – confidence interval; TGF beta 1 – t*ransforming growth factor beta* 1.

The presence of allele A in C-1260A of CYP27B1 polymorphism and CG genotype for 174 G/C of IL-6 increase the risk of a higher class according to NYHA, and a lower level of cystatin C, higher TGF beta 1 and higher eGFR MDRD decrease the risk of significant limitation during activity.

## Discussion

### The significance of selected SNPs in patients with diastolic heart failure

The human IL-6 gene is located on chromosome 7p21, and consists of 5 exons and 4 introns. IL-6 has several polymorphisms in the promoter region^5^. Conflicting results have been reported regarding the IL-6–174 G/C polymorphism and diseases. Two studies have shown that subjects who are homozygous for the G allele or heterozygous for G/C at position -174 have higher plasma IL-6 levels and higher IL-6 gene transcriptional activity than individuals homozygous for the C allele^6, 7^. However, another study reported that the CC genotype was associated with cardiovascular events in dialysis patients^8^.

Also patients undergoing coronary artery bypass graft surgery had comparable baseline IL-6 levels across different IL-6 genotypes, but, after the procedure, the rise in IL-6 was greater in carriers of the CC genotype than in carriers of the G allele^9^.

In our study, in the group of hypertensive patients with diastolic HF, there were significantly more patients with the G allele, which, however, was not associated with significant differences in the levels of IL-6. These findings point to a difference in IL-6 production in response to pathologic stimuli that is genetically determined^10^. The aim of the study of Weng *et al*. was to evaluate several candidate gene polymorphisms for their association with the risk of developing post-transplantation metabolic syndrome and diabetes mellitus (PTDM)^10^. The authors observed that patients with the IL-6 G/G genotype experienced a lower risk of developing PTDM, and after adjusting for different variables, confirmed this observation (OR, 0.05; 95% CI, 0.00–0.66) and concluded that G/G genotype of IL-6 may play an important role in lowering the risk for PTDM development^10^. In another study on the other hand the hemodialysis patients with the genotypes for the proinflammatory cytokine IL-6 (G/G and G/C) had significantly higher comorbidity and lower functional scores (Karnofsky index) compared with patients with the CC genotype^11^.

In our study GG homozygotes in 174 G/C of IL6 polymorphism were characterized by higher values of GFR MDRD compared to the non-GG profile. Perhaps the GG profile in 174 G/C polymorphism is protective in hypertensive patients with HFpEF or HFmrEF but may be harmful in patients with end stage renal disease.

Arterial stiffness and a decrease in systolic annular mitral velocity are often observed in HFpEF. Sie *et al*. found pulse wave velocity to be 0.35 m/s higher for CC-homozygotes vs. wildtype GG-homozygotes (p = 0.018) with evidence for an allele-dose effect (p trend = 0.013), with a similar pattern for pulse pressure (p trend = 0.041). The interleukin-6-174 GG genotype was associated with lower arterial stiffness and pulse pressure^12^.

In our study GG homozygotes had higher systolic mitral annular velocities corresponding to better function of longitudinal fibers of myocardium.

Another polymorphism associated with the inflammatory state assessed by us was TNF alpha 308 (rs1800629) polymorphism. In patients with rheumatoid arthritis carriers of the minor allele A in TNF alpha 308 (rs1800629) polymorphism exhibited a higher risk of CV events after adjustment for demographic and traditional CV risk factors (p = 0.023, HR 1.72 [95% CI 1.076–2.74])^13^. The maternal blood pressure in the third trimester was significantly higher in the group of women possessing the TNF2 (A) allele compared to homozygous for the TNF1 (G) allele (systolic BP, p < 0.01 and diastolic BP, p < 0.05). The maternal TNF2 (A) allele of the TNF-alpha promoter region at position -308 could play a role in the alteration of blood pressures^14^. The analysis of Bolotskykh *et al*. showed that carrying the A allele was significantly associated with moderate stage of LV diastolic diameter, left atrial volume, a history of myocardial infarction, body mass index and diastolic blood pressure^15^.

However, when HFpEF patients were stratified on the basis of risk factors such as diabetes mellitus, hypertension and obesity, TNFa 308 G/A polymorphisms did not modulate the risk of LV dysfunction due to these factors. Based on the above findings the authors indicated TNF alpha 308 G/A and A/A genotypes as risk factors for ventricular remodeling and LV dysfunction progression^15^. In our study in hypertensive patients with diastolic HF, carrying allele A in TNF-308 G/A rs1800629 polymorphism was significantly associated with higher incidence of paroxysmal atrial fibrillation and could also play a role in the alteration of blood pressure.

LV hypertrophy increases the risk of cardiovascular morbid events in hypertension. TGF-beta 1 is involved in pathologic states such as cardiac hypertrophy and cardiac fibrosis. In our study CC genotype of TGF beta 1 was correlated with LV mass and a higher concentration of the proinflammatory cytokine TNF alpha in plasma. There are only a few studies on TGF beta rs 1800471 polymorphism. Kiliś-Pstrusiństa *et al*. found no relationships between rs1800471 polymorphism allele transfer and the incidence or progression of non-diabetic chronic kidney disease, but they found that the serum TGF-beta 1 was significantly higher in patients than in controls. Circulating TGF-beta 1 level is determined in a multifactorial way, not by rs1800471 polymorphism in the TGFB1 gene^16^.

In our study we made a similar observation: in hypertensive patients with heart failure there was no significant correlation between assessed TGF beta 1 polymorphism and TGF beta 1 concentration in plasma. Morris DR *et al*. in their meta-analysis revealed that common genetic polymorphisms in TGF-β1 such as rs1800471 are associated with complications of coronary heart disease^17^. In the study of Xu *et al*. 680 essential hypertensive patients were divided according to the presence of LV hypertrophy (LVH). For + 915 Arg– > Pro at codon 25, the LV mass index in Arg-Pro genotype carriers was significantly higher than in the Arg-Arg and Pro-Pro carriers^18^. Multivariate analysis showed that the Arg-Pro genotype was an independent risk factor for LVH (OR 3.23, 95% CI [1.48–5.63, P = 0.002]). The study of Xu *et al*. as well as ours revealed a genetic association of TGF-beta1 + 915 Arg– > Pro at codon 25 polymorphism with LVH or LV mass in the hypertensive population^18^.

The next assessed polymorphism (-786 T/C NOS rs2070744) is involved in endothelial function. This polymorphism located in the promoter region is associated with regulation of NOS3 gene expression level. Carriers of the T allele have lower levels of nitric oxide than CC homozygotes^19^. Polymorphism of the endothelial nitric oxide synthase (eNOS) gene may be implicated in the development of nephropathy. T(-786)– > C polymorphism may be involved in the progression of both nondiabetic and diabetic nephropathy, along with intron 4 polymorphism. In the study of Asakimori *et al*. the frequencies of the T/C and C/C genotypes were significantly higher in the nondiabetic hemodialysis patients than in the controls (odds ratio 1.41; 95% CI 1.03–2.00), and were also significantly higher in the diabetic hemodialysis patients than in the controls^20^. Zanchi *et al*. examined four eNOS polymorphisms, and two were associated with diabetic nephropathy in the case-control comparisons: a T to C substitution in the promoter at position -786 and the a-deletion/b-insertion in intron 4^21^. It has been proposed that an increase in oxidative stress in HF leads to a decrease in nitric oxide signaling, in turn leading to impaired nitroso-redox signaling. In the study of Kose M. *et al*.^22^, endothelial dysfunction was present in congestive HF, and the presence of endothelial nitric oxide synthase promoter polymorphism (thymidine to cytosine transition [T(-786)C]) further impaired endothelium-dependent vasodilation. In addition to its well-recognized role in the regulation of vascular tone, nitric oxide modulates sympathetic and parasympathetic nervous system activities. Abnormalities of both autonomic control and nitric oxide synthase activity are known to occur in patients with congestive HF^22^. Recently, a polymorphism of the promoter of the endothelial nitric oxide synthase (eNOS) gene has been associated with a reduction of eNOS activity. Patients homozygous for the polymorphism of the eNOS promoter had a greater autonomic imbalance as reflected by significant differences in high- and low-frequency heart rate variability, so this polymorphism may serve as a marker for patients at increased risk for sudden death and more rapid progression of disease^23^. In the Genetic Risk Assessment of Heart Failure (GROH) substudy of the African-American Heart Failure Trial, in 786 T/C promoter NOS3 polymorphism, the T allele was associated with LVEF (p = 0.01). In HF subjects, NOS3 genotype influences blood pressure and LV remodeling^24^.

In our study patients with the C allele in NOS polymorphism had a higher class according to the NYHA classification, lower blood pressure and LVEF and worse parameters of kidney function (cystatin C and eGFR MDRD) compared to patients without the C allele.

In our study patients with the AA profile in C-1260A of CYP27B1 polymorphism had lower values of eGFR (MDRD) and higher levels of uric acid compared to CA and CC profiles. Vitamin D and its analogues are reported to have renoprotective effects in chronic kidney disease including diabetic nephropathy (DN). Vitamin D(3) is converted to 1,25(OH)D(3) by CYP2R1 and CYP27B1. The biological action of 1,25(OH)D(3) is mediated via its receptor. In the study by Martin *et al*. no significant differences were observed in genotype or allele frequencies between case and control groups for VDR, CYP27B1 or CYP2R1 SNPs, either before or after stratification by the recruitment centre or when restricted to patients with end-stage renal disease^25^. Patients with gout have lower calcitriol levels that improve when uric acid is lowered. The mechanism of these observations is unknown. In the study of Chen W. *et al*. hyperuricemia suppresses 1-α-hydroxylase, leading to lower 1,25(OH)2D and higher parathormone in rats, and this is mediated by nuclear factor kappa-light-chain-enhancer of activated B cells (NFκB)^26^.

### TGF-beta 1 as the biochemical independent risk factor for worse heart failure course based on the NYHA classification

TGF-beta 1 is one of three isoforms of the TGF-beta superfamily. Transforming growth factor beta 1 (TGF-beta 1) represents a central regulator of cardiac fibrosis^27–29^. Alterations in the structure of cardiac tissue, particularly fibrous tissue transformation, are considered to be the major cause of cardiac remodeling. The accumulation of extracellular matrix increases myocardial stiffness and consequently impairs contractile behavior of the heart muscle. Regarding the role of TGF beta 1 in HF, the results are not consistent between studies^27–29^.

In the study of Behnes M. *et al*. the authors investigated serum levels of TGF-beta 1 in 401 patients with atrial fibrillation and congestive HF. Patients with HF had lower TGF-beta 1 levels than those without (p = 0.0005)^30^. Similarly, in our previous study patients with hypertension and HF had lower TGF-beta levels than those with only hypertension. In this study we revealed that hypertensive patients with HFpEF and HFmrEF and higher concentrations of TGF beta 1 were characterized by lower classes according to the NYHA classification. The lower levels of TGF beta 1 in patients with more aggravated HF may result from higher consumption of TGF-beta 1 within the impaired myocardium or antifibrotic functions of natriuretic peptides.

This supports experimental data from the literature, in which natriuretic peptides have been shown to suppress adverse structural remodeling in the atria and ventricles. TGF beta 1 is upregulated by Ang II, and induction of TGF beta 1 causes cardiac fibrosis. The interplay between Ang II and TGF beta 1 causes continued activation that may result in chronic hypertension and progressive myocardial fibrosis, leading to HF^31^.

Current HF therapies mainly focus on blocking the detrimental effects of long-term neurohormonal activation and largely ignore the physiological compensatory effect of the natriuretic peptide system and other endogenous vasodilator systems.

We hypothesized that a higher level of TGF beta might be present during earlier phases of diastolic HF and that is why it is present in patients with less severe HF symptoms.

### Heart failure with preserved and mid-range ejection fraction and kidney function

Renal dysfunction is common in patients with HF and is associated with high mortality. This relationship is well established in HF and reduced ejection fraction (HFrEF), but it is not fully understood in HFpEF and HFmrEF. In the new paradigm for HFpEF, proposing a sequence of events leading to myocardial remodeling and dysfunction in HFpEF was recently introduced, involving inflammatory, microvascular, and cardiac components. The kidney might play a key role in this systemic process. Renal impairment causes metabolic and systemic derangements in circulating factors, causing an activated systemic inflammatory state and endothelial dysfunction, which may lead to cardiomyocyte stiffening, hypertrophy, and interstitial fibrosis via cross-talk between the endothelium and cardiomyocyte compartments^32^. In the patients admitted for decompensated HF in the RICA registry, renal dysfunction was frequently associated with HFpEF. eGFR below normal was strongly associated with all-cause mortality. Further decline of renal function was frequent, especially among diabetic and patients treated with MRA agents^33^. Also in the study of Ter Maaten *et al*. impaired renal function was a risk factor for developing HFpEF^32^. In the study of Primessnig *et al*. the rat model of chronic kidney disease (CKD) with HFpEF was associated with slowed relaxation of LV cardiomyocytes^34^.

Unger *et al*. prospectively studied 299 patients enrolled in the Northwestern University HFpEF Program^35^. CKD and reduced eGFR were both associated with worse cardiac mechanical indices including left atrial (LA) reservoir strain, LV longitudinal strain, and right ventricular free wall strain, even after adjusting for potential confounders, including co-morbidities, EF, and volume status^35^. Reduced eGFR was also associated with worse outcomes [adjusted hazard ratio (HR) 1.28, 95% confidence interval (CI) 1.01–1.61 per 1-SD decrease in eGFR; P = 0.039]^35^. Gori *et al*. studied 217 participants from the PARAMOUNT study with HFpEF who had echocardiography and measures of kidney function^36^. Renal dysfunction was associated with abnormal LV geometry (defined as concentric hypertrophy, or eccentric hypertrophy, or concentric remodeling), lower midwall fractional shortening (MWFS), and higher NT-proBNP^36^. Compared with patients without renal dysfunction, those with low eGFR and no albuminuria had a higher prevalence of abnormal LV geometry and lower MWFS^36^. In these studies in HFpEF patients, CKD was independently associated with worse cardiac mechanics, which may explain why HFpEF patients with CKD had worse outcomes^35, 36^.

Cystatin C is a marker of renal function that also predicts cardiovascular outcome. The serum cystatin C level in patients with HFpEF is an independent predictor for all-cause mortality and/or readmission in patients with acute HF, regardless of renal function^37^. Serum cystatin C is a novel and stable biomarker not influenced by sex, age, exertion, diet, body mass index, muscle mass or serum creatinine^37^]. Cystatin C is a small, low-molecular weight protein from the group of cysteine proteinase inhibitors. It is produced by all nucleated cells in the body and secreted into the extracellular space at a steady rate. With low molecular weight and a high isoelectric point, it readily undergoes glomerular filtration. In the proximal tubule it is absorbed and then catabolized, and therefore does not return to the circulation^38^. Plasma CysC has a significant advantage over other markers clinically used to estimate GFR. Impaired renal function is an independent marker for left ventricular hypertrophy (LVH) and a good predictor of morbidity and mortality in cardiovascular disease. In the study of Li *et al*. there was a positive correlation between serum cystatin C levels and interventricular septal thickness, posterior wall thickness, and LV weight index, and the serum level of cystatin C was an independent marker for hypertensive LVH^38^.

Moran *et al*. examined 4453 subjects aged 65 years or older without HF et baseline from the Cardiovascular Health Study. They compared the association of cystatin C with risk of incident HF with normal ejection fraction (HFnEF) and HF with reduced ejection fraction (HFrEF). During 8 years of follow-up, 167 cases of incident HFnEF and 206 cases of incident HFREF occurred. Increased risk of HFnEF was apparent only in the highest cystatin C quartile (HR 2.25; 95% CI 1.33–3.80), while a linear trend was present for HFrEF ^39^. In our study worse kidney function assessed with both eGFR and cystatin C was independently associated with worse course of HF with preserved and moderate reduced EF, which is consistent with the results of the above publications.

### Genetic polymorphisms independently associated with limitation during physical activity in HF patients

Vitamin D or its bioactive metabolite, 1,25 (OH)_2_ vitamin D_3_ [1,25(OH)_2_D_3_], is a nuclear hormone receptor ligand, which is known to have profound effects on calcium and phosphate homeostasis. The heart is particularly noteworthy in that plasma 25(OH) vitamin D_3_ [25(OH)D_3_] levels have been shown to correlate inversely with the incidence of a variety of cardiac disorders, including ischemic heart disease and HF. Interventional studies in a variety of cell culture systems and animal models suggest that the liganded VDR can exert antihypertrophic activity in cardiac myocytes *in vitro* and *in vivo* ^40^. Cardiac ventricular tissue also expresses 1α-hydroxylase (Cyp27B1), implying that it has the capacity to produce 1,25(OH)_2_D_3_, the cognate ligand of the VDR, from circulating 25(OH)D_3_.

Low serum levels of 25-hydroxyvitamin D3 are known to be associated with increased mortality, and several studies have reported that vitamin D3 supplementation improves cardiovascular outcomes in patients with chronic kidney disease (CKD).

However, the effects of 1,25 dihydroxyvitamin D3 [1,25(OH)2D3] on cardiovascular disease remain unclear. In the study of Ito H. *et al*. the serum levels of 1,25(OH)2D3 correlated significantly with transmitral to early diastolic mitral annular velocity ratio (E/e’) used as a surrogate marker of diastolic function and the radial augmentation index (rAI) used as an indicator of arterial stiffness and reflects LV afterload (r = −0.36, p < 0.0001; r = −0.37, p < 0.0001)^41^. Even in hypertensive patients without CKD, it was a useful indicator for LV diastolic dysfunction^41^. The genetic variation in vitamin D-dependent signaling might be associated with congestive HF in human subjects with hypertension. In the context of hypertension, an SNP in CYP27B1 was associated with congestive HF (odds ratio: 2.14 for subjects homozygous for the C allele; 95% CI: 1.05–4.39). Based on the study by Wilke RA, genetic variation in vitamin D biosynthesis is associated with increased risk of HF^42^. Our results, corresponding with the conclusions of Ito *et al*. and Wilke *et al*., show that the A allele in C-1260A of CYP27B1 was an independent predictor of worse course of diastolic HF, confirming the association of vitamin D homeostasis with LV diastolic function in hypertensive patients^42^.

The continued lack of effective therapies to improve outcomes in HFpEF underscores the knowledge gaps regarding the pathophysiology of HF with diastolic dysfunction. The new HFpEF paradigm proposes that comorbidities drive structural and functional remodeling through systemic endothelial inflammation^43, 44^.

The systemic proinflammatory state triggers a downstream cascade of cardiac microvascular endothelial activation, oxidative stress, and abnormal myocardial cyclic guanosine monophosphate signaling, leading to microvascular rarefaction, chronic ischemia, fibrosis and progression to HF^45^. Age, increased BMI, diabetes, low ejection fraction, left main stenosis, and genetic variation in the IL-6 promoter were established as significant independent risk factors for the survival of patients with three-vessel disease^46^.

Compared to those with the GG genotype, men carrying the -174C allele had a relative risk of coronary heart disease of 1.54 (95% CI 1.0–2.23, p = 0.048) and this effect was greatest in smokers^47^. These results suggest that, at least in part, the effect of the IL-6 -174G > C polymorphism on coronary heart disease is likely to operate through inflammatory mechanisms, but the genotype effect on diastolic HF is largely unexplained. In our study the -572 G/C genotype of IL-6 may be a genetic marker of diastolic HF^47^.

The promise of pharmacogenetics lies in its potential to identify the right drug at the right dose for the right individual. Many cells that play an important role in the cardiovascular system express the vitamin D receptor (VDR) and respond to 1,25-(OH)2D with cell-specific function and gene regulation. These cells include cardiomyocytes, vascular endothelial cells, vascular smooth muscle cells, phagocytes, and cells of the nephron, which produce renin.

Vitamin-D deficient patients present a higher risk of cardiovascular disease than the general population. In mice lacking CYP27B1 (1α-hydroxylase - an enzyme, which converts vitamin D to its active form), in addition to the expected phenotype (hypocalcaemia, secondary hyperparathyroidism and osteomalacia), development of hypertension and cardiac hypertrophy were also observed^48^. Moreover, these mice presented with overexpression of renin and atrial natriuretic peptide^48^. Due to the high prevalence of hypovitaminosis D among patients with high cardiovascular risk, vitamin D supplementation therapy may be warranted in this population. The meaning of the allele A in C-1260A of CYP27B1 in active vitamin D deficiency needs further investigations.

The presence CG genotype for 174 G/C of IL-6 in patients with hypertension may serve as a predictor of diastolic heart failure with inflammatory background and motivate to start with also non-pharmacological therapies reducing inflammation like smoking cessation and regular physical training. The meaning of anti-inflammatory drugs in primary hypertension needs further investigations.

## Material and Methods

### Study Population

110 patients with HF recognized based on the ESC guidelines 2012 (mean age 63 ± 11 years, 69% males) were consecutively included in the study between January 2014 and October 2015. Based on ESC guidelines 2016 our HF group contains patients with heart failure with preserved and mid-range heart failure (HFpEF and HFmrEF)^49^.

The exclusion criteria were as follows: unstable hypertension, New York Heart Association (NYHA) class IV HF, evidence of pulmonary hypertension on echocardiography, obstructive or restrictive pulmonary disease, hyperthyroidism and hypothyroidism, pregnancy and lactating, hemodynamically significant acquired heart defects with the exception of mitral and tricuspid incompetence secondary to left and/or right ventricular dilatation, cancer, significant anemia, abuse of alcohol or drugs, chronic inflammatory and other diseases, operation or severe injury during a month prior to blood collection or lack of informed consent to participate in the study. All enrolled patients underwent blinded adjudication by cardiologists experienced in adjudication. Detailed clinical, biomarker and imaging data were collected at the time of enrollment and echocardiograms were performed and interpreted by cardiologists blinded to genetic and biomarkers analysis. Functional polymorphisms were selected from six candidate genes: CYP27B1, NOS3, IL-6, TGF beta, TNF alpha, and PPAR gamma. We also assessed the levels of selected HF biomarkers and performed echocardiographic examinations.

Fasting venous blood samples were drawn in the morning and the obtained serum was frozen at the temperature of −70 °C. Estimated glomerular filtration rate (eGFR) was calculated using the Modification of Diet in Renal Disease (MDRD) formula^50^. Systolic and diastolic arterial pressures were measured using a sphygmomanometer and stethoscope.

Approval from the Bioethics Commission of the Medical University of Lodz (No. RNN/496/11/KB) was obtained. All methods in this study were performed in accordance with the guidelines and regulations approved by the Bioethics Commission of the Medical University of Lodz. Written informed consent was obtained from all the patients.

### Single nucleotide polymorphisms

In the current study, we isolated DNA from whole blood using a commercial kit (Chemagen). The approximate yields are 30–40 ng DNA/100 μl whole blood. The DNA was stored at 4 °C until required as a template in polymerase chain reaction. The polymorphisms were studied using the RFLP-PCR method. Primers and restriction enzymes are described in Table 6.

**Table 6:** Primers and restriction enzymes.

Polymorphism	Primers	Enzyme
−174 G/C IL-6 rs1800795	F 5′ GTC AAG ACA TGC CAA AGT GCT 3′	NlaIII
	R 5′ GAG GGG CTG ATT GGA AAC C 3'	
CYP27B1 -1260C/A rs10877012	F 5′ CAA AAA TTA GCC AGG CAT GGT G 3′	HinfI
	R 5′ CCT TCA ATT CCA GAA CTT CAG AGC 3'	
−786 T/C NOS rs2070744	F 5′ GAC CCC TGT GGA CCA GAT 3′	NaeI
	R 5′ CAT TCA GTG ACG CAC GCT TC 3'	
Arg25Pro TGFβ rs1800471	F 5′ CAC ACC AGC CCT GTT CGC 3′	BglI
	R 5′ CTT CCG CTT CAC CAG CTC CAT 3'	
TNF-608 G/A rs1800629	F 5′ GGC AAT AGG TTT TGA GGG cCA 3′	NcoI
	R 5′ CCT TCT GTC TCG GTT TCT TCT CC 3'	
Pro12Ala PPARγ rs1801282	F 5′ CAA GCC CAG TCC TTT CTG TG 3′	HpaII
	R 5′ AGT GAA GGA ATC GCT TTC cG 3′	

**Figure 1:**
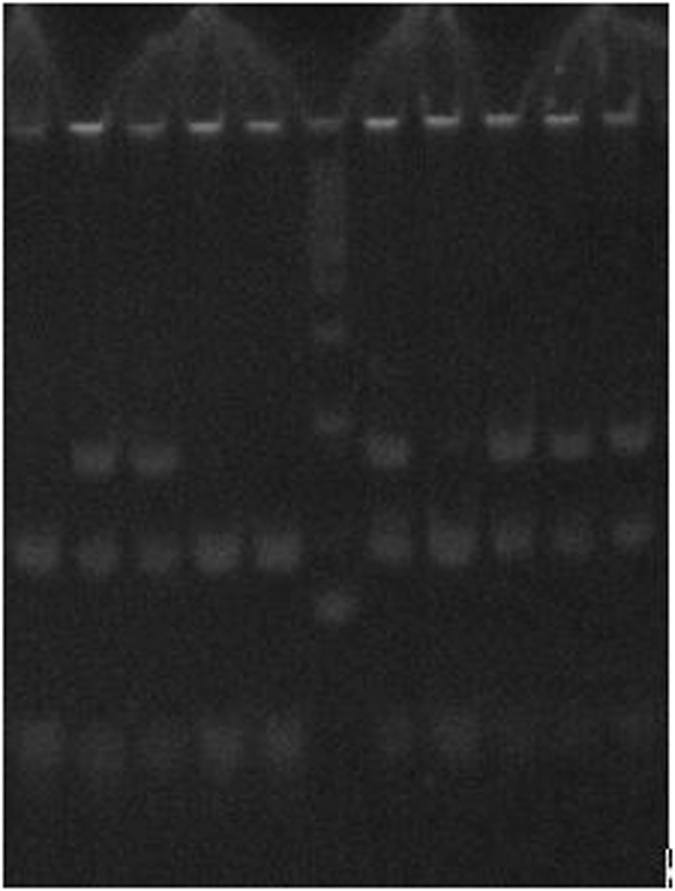
Electrophoresis on 6% polyacrylamide gel for -1260C/A CYP27B1 gene polymorphism (rs10877012). Bans on the gel represent the following genotypes: homozygous CC (118 bp + 60 bp), heterozygous CA (179 bp + 118 bp + 60 bp), homozygous AA (179 bp). The visualization was conducted with the use of ethidium bromide. The photography was made using PhotoDoc-IT Imaging System (Ultra-Violet Products Ltd, UK).

The samples after PCR reaction were digested by using appropriate enzymes and were subjected to 6% acrylamide gel and stained with ethidium bromide. Fragments for wild type, heterozygous and mutants are presented in Table 7. In Fig. 1 we present examples of single nucleotide polymorphism analysis.

**Table 7:** Fragments for wild type, heterozygous and mutants.

Polymorphism	Wild type	Heterozygous	Mutant
−174 G/C IL-6 rs1800795	173, 11	173, 122, 51, 11	122, 51, 11
CYP27B1 -1260C/A rs10877012	118, 60	179, 118, 60	179
−786 T/C NOS rs2070744	190, 68	259, 190, 68	259
Arg25Pro TGFβ rs1800471	142, 103, 60	163, 142, 103, 60	163, 142
TNF-608 G/A rs1800629	177, 19	197, 177, 19	197, 19
Pro12Ala PPARγ rs1801282	217, 20	237, 217, 20	237

### Biomarker tests

The concentrations of NT-proBNP, cardiotrophin-1 (CT-1), cystatin C (CysC), tumor necrosis factor alpha (TNF-α), collagen III N-terminal propeptide (PIIINP), syndecan-4, interleukin-1 receptor-like protein 1 (IL1RL1), transforming growth factor beta (TGF-β1) and lipocalin-2/NGAL were determined using the EMax Endpoint ELISA Microplate Reader analyzer (Molecular Devices; Sunnyvale, California, USA). TNF-α was analyzed with the enzyme-linked immunosorbent assay (Diaclone/Gen-Probe, USA), with 2 polyclonal antibodies directed against TNF-α. Determination of NT-proBNP and CT-1 was performed with reagents of USCN Life Science Inc./Cloud-Clone Corp (Wuhan, China), using a sandwich ELISA assay according to the manufacturer’s protocol. Measurement of CysC was performed using a sandwich enzyme immunoassay (BioVendor, Brno, Czech Republic) developed for the quantitative measurement of this marker in human serum. Analysis of the concentration of PIIINP, syndecan-4 and IL1RL1 was performed with a USCN Life Science Inc./Cloud-Clone Corp (Wuhan, China) kit, using a sandwich ELISA assay according to the manufacturer’s protocol. Measurement of TGF-β1 was performed using a sandwich enzyme immunoassay (Gen-Probe Diaclone SAS, Besançon, France) designed for the quantitative detection of TGF-β1 levels in cell culture supernatants, human serum, plasma or other body fluids. Determination of lipocalin-2/NGAL was conducted using the BioVendor Human Lipocalin-2/NGAL ELISA sandwich enzyme immunoassay. Analysis of the concentration of galectin-3 (GAL3) was performed with a USCN Life Science Inc./Cloud-Clone Corp (Wuhan, China) kit, using a sandwich ELISA assay.

### Echocardiography

All patients were examined following a standardized protocol using an ALOKA Alpha 10 Premier (Tokyo, Japan) with a 3–11 MHz probe. Quantitative echocardiography was used following current guidelines^51^. LV volumes and EF were determined by biplane Simpson’s method. The ratio of early (E) filling peak velocity to early diastolic (E’) mitral annular myocardial velocity was calculated as an index of LV filling pressure^51^.

### Statistical analysis

The STATISTICA 10 software package (StatSoft, Poland) and SPSS Statistics 21 (IBM, United States) were used for analysis. All values are presented as the median and 1^st^ and 3^rd^ quartile (Q25-Q75) for numerical variables and the number and percentages for categorical variables. The Shapiro-Wilk test was used to assess the normality of variables distributions.

To examine the relationship between qualitative variables the chi-square test of independence or chi-square test with Yates’ correction and maximum likelihood chi-square test were used. To compare two independent groups, Student’s t-test for continuous variables with normal distribution and the Mann-Whitney U test for non-normally distributed variables were used.

To compare more than two independent groups, analysis of variance (ANOVA, for normally distributed variables) or Kruskal-Wallis test (if the distributions of variables were different from normal) with post hoc multiple comparisons was used.

Stepwise logistic regression was used to identify factors significantly associated with the presence of significant symptoms according to the NYHA classification. A value of p < 0.05 was considered significant.

### Limitations of the Study

The study included a relatively small number of patients, and the findings need to be confirmed in a larger population. The present study was conducted as a prospective, consecutive recruitment of patients with heart failure with preserved and mid-range ejection fraction. There was only one patient with AA profile of NOS3 -786 C/T rs2070744 polymorphism and one patient with AA homozygote in TNF-308 G/A rs1800629.

## Conclusions

These data confirm the importance of the selected SNPs in aggravation and complications of hypertension. The CG genotype for 174 G/C of IL-6 and allele A in C-1260A of CYP27B1 are SNPs independently associated with worse course of HFpEF and HFmrEF.
